# Removal of Phenol from Synthetic and Industrial Wastewater by Potato Pulp Peroxidases

**DOI:** 10.1007/s11270-015-2517-0

**Published:** 2015-07-11

**Authors:** Katarzyna Kurnik, Krzysztof Treder, Monika Skorupa-Kłaput, Andrzej Tretyn, Jarosław Tyburski

**Affiliations:** Chair of Plant Physiology and Biotechnology, Nicolaus Copernicus University, Lwowska 1, 87-100 Toruń, Poland; Laboratory of Molecular Diagnostic and Biochemistry, Department of Potato Protection and Seed Science in Bonin, Plant Breeding and Acclimatization Institute-National Research Institute, 76-009 Bonin, Poland; Centre for Modern Interdisciplinary Technologies, Nicolaus Copernicus University, Wileńska 4, 87-100 Toruń, Poland

**Keywords:** Bioremediation, Peroxidase isoenzymes, Phenol, Potato pulp, Wastewater treatment

## Abstract

Plant peroxidases have strong potential utility for decontamination of phenol-polluted wastewater. However, large-scale use of these enzymes for phenol depollution requires a source of cheap, abundant, and easily accessible peroxidase-containing material. In this study, we show that potato pulp, a waste product of the starch industry, contains large amounts of active peroxidases. We demonstrate that potato pulp may serve as a tool for peroxidase-based remediation of phenol pollution. The phenol removal efficiency of potato pulp was over 95 % for optimized phenol concentrations. The potato pulp enzymes maintained their activity at pH 4 to 8 and were stable over a wide temperature range. Phenol solutions treated with potato pulp showed a significant reduction in toxicity compared with untreated phenol solutions. Finally we determined that this method may be employed to remove phenol from industrial effluent with over 90 % removal efficiency under optimal conditions.

## Introduction

Phenol and its derivatives are major organic pollutants, produced by various industrial activities, such as coal conversion, petroleum refining, resin, plastic and pharmaceutical production, wood preservation, metal coating, and textile dyeing (Michałowicz and Duda 2007; Hamid and Rehman 2009; Varsha et al. 2011). Moreover, pesticide production and degradation release huge quantities of phenols into the environment (Michałowicz and Duda 2007).

Exposure to most phenolic compounds poses a serious risk to human health, due to their toxic, mutagenic, carcinogenic, and teratogenic effects (Michałowicz and Duda 2007). Therefore, phenolic compounds need to be eliminated from wastewater before the water is discharged into the environment (Hamid and Rehman 2009). Conventional, physico-chemical methods for phenol removal from industrial wastewaters include membrane filtration; coagulation/flocculation; ion exchange; electrolysis, adsorption on activated carbon; advanced oxidation processes (chlorination, bleaching, ozonation, Fenton oxidation, photocatalytic oxidation); and chemical reduction. All these methods suffer from serious shortcomings due to limited effectiveness and high operational costs (Radushev et al. 2008; Qayyum et al. 2009). Thus, considerable work has aimed to develop alternative methods for the removal of phenolic pollutants from industrial waste streams and the environment. Biodegradation has emerged as one of the most promising approaches for processing water contaminated with phenols. Several reports show partial or complete biodegradation of such chemicals by cultures of bacteria, fungi, or algae (Aitken et al. 1989; Longoria et al. 2008). However, using microbes for treating pollutants is still an expensive process, due to the high costs of producing the microbial culture. Moreover, inhibitors present in the effluent may suppress growth of the culture and the expression of enzymes involved in the degradation of phenolic compounds may vary over time, or depend on the growth phase of the organism (Qayyum et al. 2009).

Enzymatic treatment with peroxidase has been proposed as another strategy for the remediation of phenol-contaminated wastewater. Peroxidases catalyze the oxidative transformation of a variety of organic and inorganic compounds at the expense of peroxide, usually H_2_O_2_. Peroxidases oxidize phenolic compounds to highly reactive radical species, which spontaneously produce insoluble oligo- or polymeric derivatives. These insoluble, polymerized products may be removed from solutions by simple filtration or sedimentation techniques (Regalado et al. 2004). Peroxidases that have been used for treatment of phenolic contaminants include fungal class II peroxidases, such as lignin peroxidase (LiP) or manganese peroxidase (MnP), or plant class III peroxidases, including horseradish peroxidase (HRP), soybean peroxidase, turnip peroxidase (Regalado et al. 2004), or bitter gourd peroxidase (Ashraf and Husain 2010). Among class III peroxidases, HRP has been most commonly used for studies of phenol removal. However, the high cost of purified HRP and its high susceptibility to inactivation by various side reactions during treatment diminish the usefulness of HRP for large-scale bioremediation (González et al. 2008). Soybean peroxidase, considered as an alternative to HRP, is cheaper than HRP, and displays some favorable characteristics, such as higher thermo-tolerance and higher conformational stability over a wide pH range. However, like HRP, soybean peroxidase is still quite susceptible to inactivation during phenol oxidation and polymerization (Feng et al. 2013).

Besides the preparations of purified enzyme (immobilized or free), several peroxidase-producing systems have been considered for use in phenol remediation. Hairy root cultures have been tested for their ability to remove phenol or dichlorophenol (González et al. 2006, 2008, 2012; Singh et al. 2006; Paisio et al. 2010; Sosa Alderete et al. 2012; Jha et al. 2013). Hairy roots offer an attractive system for this purpose due to their ability to produce large quantities of exudates that are rich in peroxidase and chelating agents. Moreover, hairy roots have fast rates of biomass production, providing an extensive area of contact with contaminated solution (Suza et al. 2008). Most studies on phenol remediation by hairy roots have been conducted on a laboratory scale. However, recent work has successfully scaled up the removal of 2,4-dichlorophenol using *Brassica napus* hairy roots in stirred tank reactor, thus opening future prospects for employing hairy roots on an industrial-scale for biodepollution of wastewater (Angelini et al. 2011).

Besides hairy root cultures, some other sources of peroxidase have been considered for utilization in wastewater decontamination. Crude enzyme extracts from *Raphanus sativus* roots efficiently remove phenolic compounds from wastewater from the leather industry (Diao et al. 2011). Juice from cut roots of *Raphanus* was also able to decontaminate phenol-polluted synthetic wastewater (Naghibi et al. 2003). The peroxidase-containing homogenate from onion solid waste, obtained after peeling the bulbs, was examined for its ability to oxidize caffeic acid, the main contaminant in wastewater from olive mills (El Agha et al. 2008). Minced shepherd’s purse (*Capsella bursa*-*pastoris*) roots were used for the remediation of soil contaminated with 2,4-dichlorophenol (Park et al. 2006).

One prerequisite for the large-scale application of plant peroxidases for bioremediation is the availability of large quantities of cheap material containing highly active, robust, and inactivation-resistant enzyme. In this work, we assess the potential use of potato pulp for phenol removal. Potato pulp is a waste product obtained during starch production. It constitutes the residue that remains after washing out nearly all the starch from potato mash (Lesiecki et al. 2012) and is composed of juice water (90 %), cellulose, hemicelluloses, pectin, starch, proteins, free amino acids, and salts (Mayer and Hillebrandt 1997; Mayer 1998). The starch manufacturing industry produces huge amounts of potato pulp, running into the hundreds of thousands of tons per year (Mayer 1998). The discharge of potato pulp has become an environmental pollution problem and a cost factor, partly due to the huge quantities of this waste product (Wang et al. 2010).

Here, we demonstrate that potato pulp, obtained from a potato starch factory, contains active peroxidases. Then, we evaluated this industrial waste for its efficiency in removing phenol and established the optimum conditions to obtain the maximum decontamination efficiency. Then we used a phytotest to evaluate the toxicity of phenol solutions before and after treatment with potato pulp. Finally, we assessed the potential use of potato pulp to reduce phenol content in the effluent from the fine mechanics industry.

## Materials and Methods

### Plant Material

Potato pulp was obtained from the starch company PPZ Trzemeszno, Poland. The raw material was portioned, frozen, and stored at −20 °C. Directly before use, the pulp was thawed in room temperature.

### Potato Pulp Peroxidase Isolation and Activity

Peroxidase (POX, EC 1.11.1.7) activity of potato pulp was assessed by measuring the rate of oxidation of pyrogallol to purpurogallin, by following the change in absorbance at 420 nm. The assay medium was composed of 100 mM phosphate buffer (pH 6.0), 39 mM pyrogallol, 23.5 mM H_2_O_2_, and 5–60 mg of potato pulp in a total volume of 1 ml. The components of the reaction mixture were mixed in a 2-ml centrifuge tube. The reaction was initiated by adding H_2_O_2_ and conducted for 1 min. After that time, it was stopped by adding H_2_SO_4_ to each tube, to a final concentration of 2 %. The A_420_ was measured immediately after adding H_2_SO_4_ to the medium, in 1-cm quartz cuvettes. Enzyme-free controls were run along with the test samples to correct the assay for non-enzymatic pyrogallol oxidation. POX activity was expressed as the micromoles purpurogallin per minute

To extract the soluble peroxidases and the peroxidases that were ionically bound to the cell wall, the samples consisting of 2.5–10 g of potato pulp were incubated for 3 h in 25 ml of McIlvaine buffer (127 mM Na_2_HPO_4_, 36.9 mM citric acid), pH 6.0, supplemented with 1 M NaCl, under constant agitation. After that, the samples were filtered through nylon mesh and centrifuged for 20 min at 10,000×g at 4 °C to remove the solid phase. The extract was dialyzed overnight against three changes of 0.25× McIlvaine buffer. Then, 30 μl of dialyzed extract was subjected to native electrophoresis on a polyacrylamide gel. The samples were subjected to discontinuous polyacrylamide gel electrophoresis under non-denaturing, non-reducing conditions, as described by Laemmli (1970) using a stacking gel containing 4 % (*w*/*v*) acrylamide and a separating gel containing 15 % (*w*/*v*) acrylamide. After the completion of electrophoresis, the gels were stained for POX activity. To visualize POX activity, the gels were placed in Pierce 1-Step TMB-Blotting Substrate Solution (Pierce) until the bands appeared. Peroxidase isoforms stained dark blue. Along with the samples, molecular mass markers (11–245 kDa, 3-Colour Prestained Protein Marker, DNA Gdańsk) were subjected to electrophoretic separation.

### Phenol Removal Conditions from Synthetic Wastewater

The reactions for phenol removal were carried out in 15-ml polypropylene tubes containing the reaction mixture composed of aqueous phenol solution, potato pulp, and H_2_O_2_, in a total volume of 2.5 ml. The reactions were incubated for 2 or 3 h at 20 ± 2 °C in an orbital shaker at 200 rpm. After incubation, the residual phenol was measured spectrophotometrically. Following the measurement, the percentage of phenol removal (removal efficiency) was calculated. If not indicated otherwise, tap water was used as the reaction medium. The stock solutions of phenol and H_2_O_2_ were prepared using distilled water.

To optimize the conditions of phenol removal, the effect of several variables, such as the weight of potato pulp inoculum, H_2_O_2_, phenol concentration, the effect of PEG 3350, pH of the reaction mixture, the incubation temperature, and shaking rate were investigated. To optimize the mass of the potato pulp inoculum, we determined the efficiency of phenol removal in reactions performed with 200, 300, 400, or 500 mg of potato pulp. The samples were added to 2.5 ml of 1 mM phenol solution and the reactions were initiated by supplementing the mixture with H_2_O_2_ to a final concentration of 2.59 mM. To determine the optimal H_2_O_2_ concentration, the mixture of 1 mM phenol solution with 400 mg potato pulp was supplemented with H_2_O_2_ to final concentrations of 1.29 to 3.24 mM. The efficiency of the removal reaction was tested within the range of initial phenol concentrations from 1 to 6 mM. The phenol solutions were mixed with 400 mg of potato pulp and then the reaction was initiated by adding H_2_O_2_ to a concentration of 2.59 mM. Simultaneously, we tested the effect of polyethylene glycol (PEG) 3350 on the efficiency of the phenol removal reaction, by supplementing the reaction mixtures with 100 mg/l PEG.

### Control Variants

To assess the involvement of the native proteins in phenol removal, we performed control reactions replacing the raw potato pulp with autoclaved potato pulp. The reactions with autoclaved material were carried out in the presence of 1–3 mM phenol and 2.59 mM H_2_O_2_. Furthermore, controls without potato pulp were performed by measuring residual phenol concentration after 2–3 h incubation of 1 to 3 mM phenol solutions in the presence or absence of H_2_O_2_. The reaction mixtures were incubated at room temperature with 200 rpm shaking.

### pH Range

To assess the effect of pH on the efficiency of phenol removal, the reactions were performed using buffered solutions of different pH values (pH 2 to 10) as the reaction medium. The reaction buffer consisted of 0.2 M acetic acid, 0.2 M phosphoric acid, and 0.2 M boric acid. Concurrently, tap water (pH 7.3) was tested. The assay was conducted as described above.

### Effect of Temperature

To assess the effect of the incubation temperature on the efficiency of phenol removal, the reactions were carried out, in reaction mixtures composed of 1–3 mM phenol, 400 mg potato pulp, and 2.59 mM H_2_O_2_, at temperatures from 10 to 60 °C, for 2 or 3 h. After the reaction was finished, residual phenol was measured spectrophotometrically.

### Effect of Shaking

Reactions were performed in optimized conditions, with shaking rate as the variable. We tested shaking rates from 0 to 250 rpm. The removal reaction was carried out in the presence of 1–3 mM phenol, 400 mg potato pulp, 2.59 mM H_2_O_2_, and incubated for 2 or 3 h. After that, the residual phenol was measured spectrophotometrically.

### Phenol Removal from Industrial Effluent

The conditions of phenol removal were essentially the same as for assay with the synthetic wastewater, except the phenol solution was replaced by industrial wastewater from fine mechanics industry of Sohbi Craft Poland. The potato pulp samples of 50 to 200 mg and 0.65 to 3.88 mM H_2_O_2_ were added to the reaction mixtures. Control assays without H_2_O_2_ were performed to estimate the peroxidase-independent phenol removal. The effect of the incubation temperature and pH on the efficiency of phenol removal was determined as described above. The efficiency of phenol removal was calculated by comparing phenol concentration in post-reaction mixture to the phenol concentration in the untreated sample.

### Phenol Determination

The phenol concentration in synthetic or industrial wastewater was measured spectrophotometrically using the 4-aminoantipyrine method, as described by Kinsley and Nicell (2000). In brief, 1 ml of post-reaction solution was mixed with 5 μl of NH_4_OH, 5 μl of 2 % 4-aminoantipyrine, and 10 μl of 8 % potassium hexacyaniferrate. After 5 min, the absorbance of the mixture was determined at 510 nm and compared with the calibration curve in the phenol concentration range of 5–40 mg/l.

### H_2_O_2_ Determination

The unreacted H_2_O_2_ remaining after the reaction was measured using 1 M potassium iodide. In brief, 500 μl of tap water and 1000 μl of 1 M KI were sequentially added to 500 μl of post-reaction solution. Then, the absorbance of the mixture was measured at 390 nm and compared with the calibration curve in the range of 0–1.75 mM H_2_O_2_.

### Garden Cress (*Lepidium sativum*) Toxicity Test

*Lepidium sativum* seeds were preincubated for 1 h in tap water and then distributed on Petri dishes lined with wet lignin. Ten seeds were placed into each plate. Thereafter, the seeds were treated with the post-reaction phenol solutions and incubated for 24 or 48 h at room temperature. As negative controls, untreated phenol solutions or phenol solutions incubated with potato pulp without hydrogen peroxide were used. For the positive control, phenol solution was replaced with water. To test the toxicity of hydrogen peroxide alone, the phenol solution was replaced with water and 0.55 mM H_2_O_2_ (the H_2_O_2_ concentration, determined to be present in the mixture after reaction when 1 mM phenol was used) was added to the mixture. After 24 or 48 h, the length of the roots of the seedlings was measured.

### Statistics

All of the experiments were conducted as three independent replicates. Mean and standard deviation were calculated. To determine whether the differences between the results were statistically significant, we performed one-way ANOVA with a significance threshold of 0.05. All the statistical analyses were performed using SigmaPlot 11.0 (Systat Software). The figures show representative results for each experiment.

## Results and Discussion

### Peroxidase Activity and Isoenzyme Analysis

**Fig. 1:**
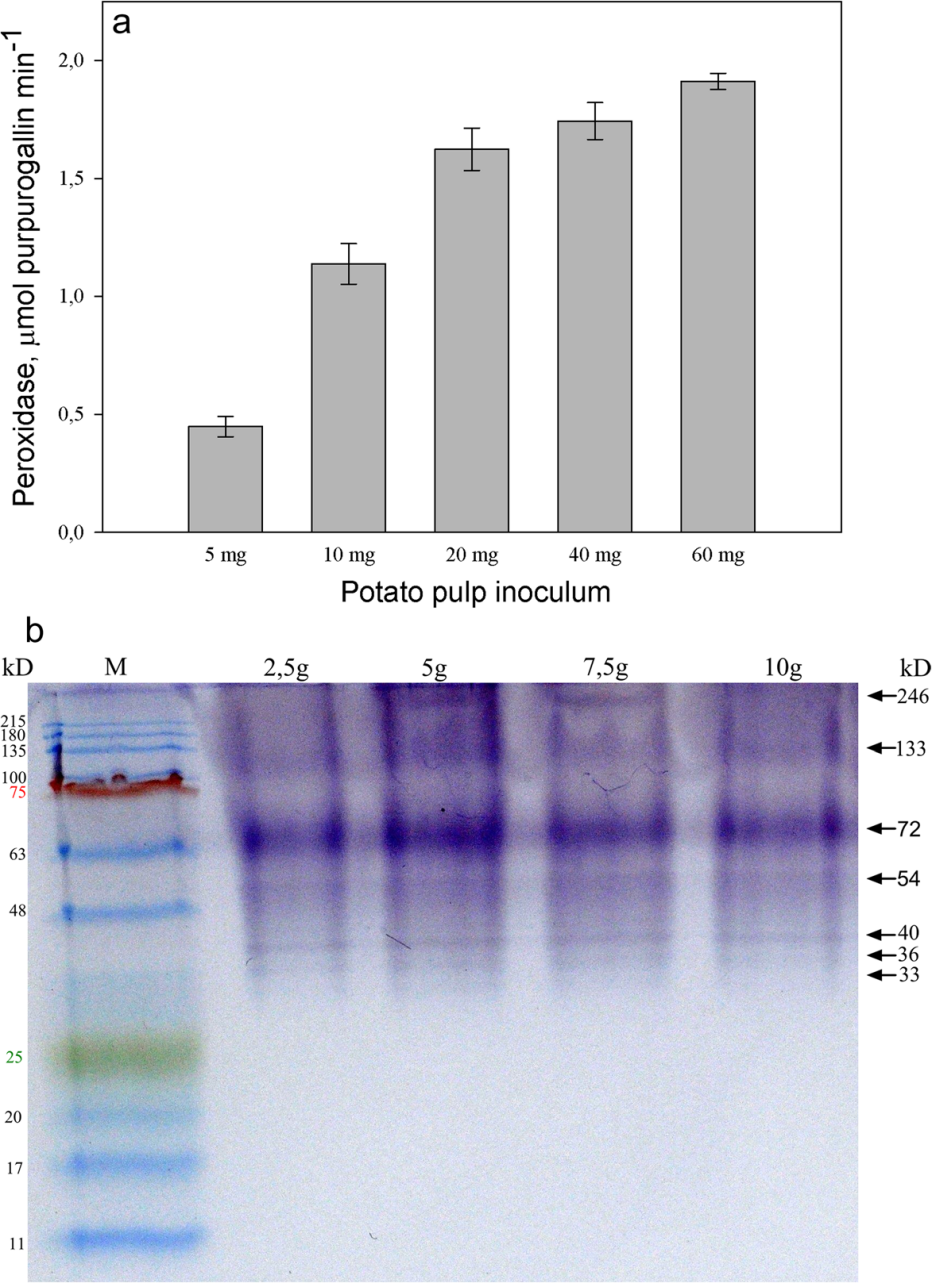
Peroxidative activity of potato pulp. A series of sample weights was tested for the ability to oxidize pyrogallol to purpurogallin in the presence of H_2_O_2_ (**a**). The isoenzyme pattern of potato pulp peroxidases. Protein preparations obtained from 2.5, 5, 7.5, or 10 g of potato pulp were separated electrophoretically and stained for peroxidase activity. Since the proteins were subjected to non-denaturing PAGE, the protein molecular mass could not be precisely determined. Therefore, the molecular masses (kDa) of peroxidase isoforms indicated represent an approximation (**b**)

The peroxidase activity of potato pulp was assayed by testing its ability to oxidize pyrogallol to purpurogallin in the presence of H_2_O_2_. Increasing the mass of the potato pulp in the reaction mixture resulted in a progressive increase in the production of purpurogallin (Fig. 1a). This finding demonstrates that the peroxidase activity is associated with potato pulp. When the protein preparation, obtained from potato pulp by extraction with NaCl-supplemented buffer, was subjected to native electrophoresis and monitored for peroxidase activity, seven peroxidase isoforms were detected. Among the peroxidase isoforms, the band corresponding to a 72 kDa protein was distinguished by its high staining intensity (Fig. 1b). However, further studies are required to determine whether the band represents a single protein or multiple polypeptides.

### Phenol Removal from Synthetic Wastewater

To test the capacity of the potato pulp to remove phenol from a water solution, 400 mg of potato pulp was suspended in 2.5 ml of phenol solution. The range of phenol concentrations tested was from 1 to 6 mM. The mixtures were supplemented with H_2_O_2_ to a final concentration of 2.59 mM. Simultaneously, a set of samples representing each phenol concentration was supplemented with PEG 3350. Reaction mixtures with and without PEG were accompanied by control samples without H_2_O_2_. The reactions were incubated at room temperature under constant agitation. The residual phenol was measured after 2 or 3 h of incubation. After both 2 and 3 h of reaction, we observed very high efficiencies of phenol removal, reaching 99 %, in reaction mixtures with initial phenol concentrations from 1 to 3 mM. These results are similar to those obtained by González et al. (2012), where phenol at concentrations of 0.1 to 2.65 mM was removed from the solution by hairy roots of *Brassica napus* with 100–80 % efficiency, respectively. However, our results show higher phenol removal efficiency than that obtained using *Raphanus sativus* roots (Naghibi et al. 2003).

**Fig. 2:**
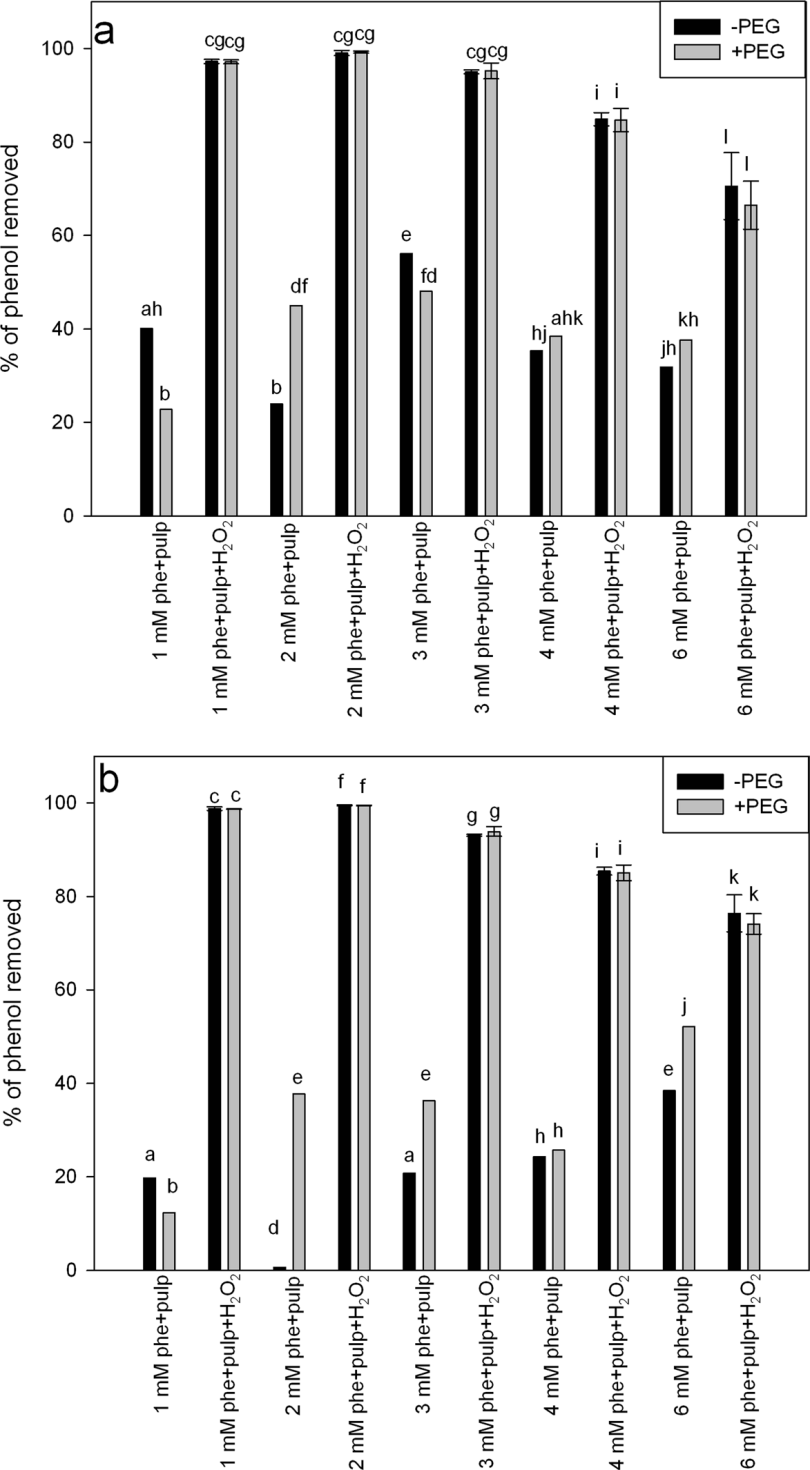
Removal efficiencies at different phenol (phe) concentrations. The efficiency of phenol removal was assessed in assay mixtures composed of phenol solution, potato pulp inoculum, and H_2_O_2_ and in corresponding control mixtures containing phenol and potato pulp but not H_2_O_2_. The efficiency of phenol removal was determined after 2 (**a**) or 3 (**b**) hours of incubation in a reaction containing 1–6 mM phenol, 400 mg potato pulp, and 2.59 mM H_2_O_2_ in tap water as a reaction medium. Reaction mixtures were either supplemented (*gray bars*) or not supplemented (*black bars*) with 100 mg/l PEG. Different *letters* denote significant differences at *p* ≤ 0.05

After 2 h of reaction, we observed no significant differences in the efficiency of phenol removal between the variants with initial phenol concentrations from 1 to 3 mM. After 3 h of incubation, we observed that the reaction mixtures with 3 mM initial phenol concentration displayed significantly lower phenol removal efficiencies than the 1 or 2 mM variants. Phenol solutions with higher concentration were degraded with significantly lower efficacy. Approximately 80 or 70 % phenol was removed from solutions with initial concentrations of 4 and 6 mM, respectively (Fig. 2a, b). However, those results still demonstrate higher removal efficiencies than those obtained by Sosa Alderete et al. (2012), who employed tobacco hairy roots to eliminate phenol from solution. The authors showed that treating 4.25 or 6.37 mM phenol, in the presence of 2.5 mM H_2_O_2_, resulted in the removal of 51.7 or 42.2 % of the pollutant, respectively (Sosa Alderete et al. 2012). The high efficiency of phenol removal by potato pulp was strongly dependent on the presence of H_2_O_2_ in the assay medium. In contrast to the reaction mixtures supplemented with H_2_O_2_, the phenol removal from H_2_O_2_-free samples did not exceed 55 %, irrespective of the initial phenol concentration. This finding strongly supports the idea that the highly efficient phenol removal is possibly due to peroxidases from the potato pulp (Fig. 2).

**Fig. 3:**
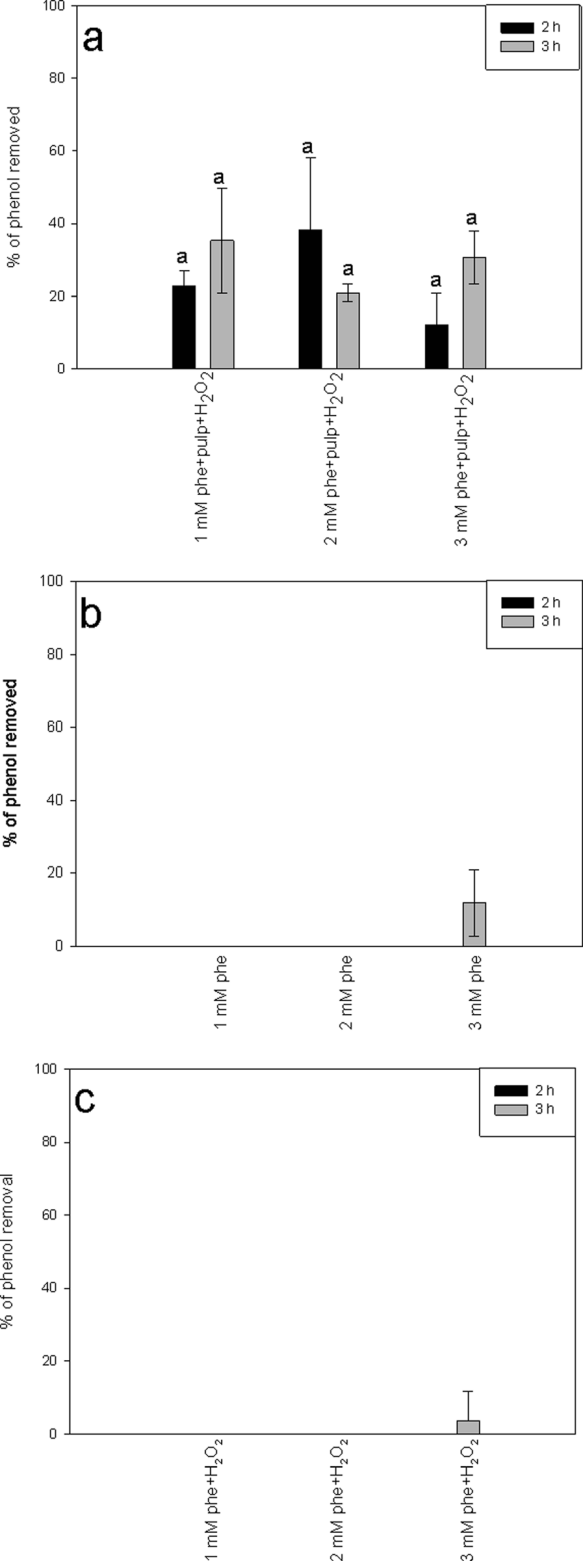
Control variants. The percentage of phenol (phe) removed after the incubation of 1–3 mM phenol solutions with autoclaved potato pulp and 2.59 mM H_2_O_2_ for 2 (*black bars*) or 3 (*gray bars*) hours (**a**). The percentage of phenol removed after the incubation of water solutions of phenol for 2 or 3 h (**b**). The percentage of phenol removed after the incubation of 1–3 mM phenol solutions supplemented with 2.59 mM H_2_O_2_ for 2 or 3 h (**c**). Different *letters* denote significant differences at *p* ≤ 0.05

To provide further evidence for the enzymatic nature of the phenol degradation process, potato pulp was autoclaved to inactivate enzymes prior to composing the reaction mixture. Phenol concentrations of 1, 2, or 3 mM were selected for this control assay, since they were the most efficiently removed if the native potato pulp was used. The reactions with the autoclaved potato pulp showed much lower removal efficiencies compared to the experiments with native pulp. In contrast to native potato pulp, the average phenol removed by the autoclaved material did not exceed 40 % (Fig. 3a, b). There were no significant differences in phenol removal efficiency between the tested initial phenol concentration variants (Fig. 3a). Partial phenol removal by autoclaved potato pulp might be caused by the adsorption of phenol on the surface of potato pulp. We also observed that the phenol removal efficiencies were negligible if the potato pulp was not added to the reaction, either in the absence or in the presence of H_2_O_2_ (Fig. 3b, c). These findings suggest that the decrease in the phenol concentration observed in our experiments was not due to spontaneous degradation and/or oxidant-dependent polymerization of a non-enzymatic nature.

Enzyme inactivation is considered to be the main disadvantage of peroxidase-based phenol removal, significantly elevating the cost of treatment (Feng et al. 2013). Three mechanisms of enzyme inactivation have been proposed as follows: (1) the reaction of an active enzyme intermediate with excess peroxide; (2) destruction of heme by strong reagents, such as free radicals generated during the enzymatic reaction; or (3) adsorption of the enzyme on the precipitated polymerization products, which occludes the enzyme’s active site (Mao et al. 2013).

The rate of enzyme inactivation may be decreased by supplementing the reaction medium with a substance that decreases the adsorption of polymers onto the enzyme’s active site, using substances such as polyethylene glycol (PEG). Several authors reported a stimulatory effect of PEG on the efficiency of phenol removal by HRP or soybean peroxidase (Kinsley and Nicell 2000). PEG substitutes for the enzyme in adsorption onto the solid polymer products, thus preventing the enzyme from co-precipitation with the polymeric products (Sakurai et al. 2003). PEG also efficiently suppressed HRP inactivation via heme destruction (Mao et al. 2013). Consequently, the addition of PEG helps to maintain peroxidase activity in the reaction mixture and can reduce the amount of the enzyme required (Qayyum et al. 2009).

When potato pulp was used in the removal process, we observed very high efficiencies of decontamination in the absence of any protective factors and the addition of PEG 3350 did not change phenol depletion at the tested concentrations (Fig. 2). Similarly, phenol removal by tomato hairy roots displayed comparable efficiency in PEG-supplemented and PEG-unsupplemented reaction mixtures. By contrast, adding PEG to the assay medium significantly increased phenol removal by rapeseed hairy roots (Paisio et al. 2010). Similarly, González et al. (2008) reported that PEG supplementation improves the efficiency of the reaction with tomato hairy roots. Wu et al. (1997), using HRP, observed the same improvement in phenol removal after supplementing the reaction medium with PEG. Also, Cooper and Nicell (1996) reported that adding polyethylene glycol resulted in a 22-fold decrease in the amount of enzyme required to effectively perform the removal reaction. The lack of difference between PEG-supplemented and PEG-unsupplemented samples observed in our experiments is consistent with the results of Caza et al. (1999), who found that adding PEG did not drastically improve the efficiency of phenol removal by soybean peroxidase.

Another approach to improve the performance of peroxidase in the detoxification of pollutants is the use of enzymes immobilized on natural or synthetic supports. Many materials and different methods, such as glass beads, magnetic beads (Bayramoğlu and Arica 2008), polymers, ion exchange resins, magnetite, aluminum-pillared clay, or sodium alginate encapsulation, have been used for peroxidase immobilization (Alemzadeh and Nejati 2009; Husain and Ulber 2011). Immobilized enzymes usually have a longer operational lifetime, being more stable to physical, chemical, and biological denaturing agents (Rao et al. 2010). Immobilization on glutaraldehyde-activated aminopropyl glass beads improved the stability of HRP and the efficiency of phenol removal (Gómez et al. 2006). HRP immobilized by bioaffinity-layering could be successfully used five times, with 100 % conversion of *p*-chlorophenol (Dalal and Gupta 2007). HRP entrapped in alginate capsules could be reused up to four times without any changes in retention activity. Media containing immobilized enzymes are more suitable when large amounts of wastewater need to be processed (Alemzadeh and Nejati 2009). A possible advantage of potato pulp as a tool for the remediation of phenol-contaminated water may be that some of the enzyme is ionically bound to cell wall polymers. Given that enzymes display improved resistance to inactivation when immobilized on a solid support, this fixation of the peroxidase to a solid fraction of the potato pulp may, at least partly, account for the robustness and stability of these enzymes in the assay medium.

### Residual H_2_O_2_

Since H_2_O_2_ is toxic for many organisms, it is necessary to determine the residual concentration of H_2_O_2_ before releasing the post-reaction mixtures into the environment (Paisio et al. 2010). We assessed the H_2_O_2_ concentration in post-reaction mixtures initially supplemented with 1, 2, or 3 mM phenol and 2.59 mM H_2_O_2_ and found, that in the post-reaction solution initially supplemented with 1 mM initial phenol, the residual H_2_O_2_ was 0.546 ± 0.09 mM. For the mixtures supplemented with 2 or 3 mM initial phenol, the residual H_2_O_2_ was present at much lower levels, 0.066 ± 0.002 or 0.020 ± 0.001 mM, respectively. Studies on H_2_O_2_ toxicity in an oligodendrocyte cell line indicated that H_2_O_2_ concentrations greater than 0.1 mM (10^−4^ M) induce cell toxicity (Bhat and Zhang 1999; Twiner et al. 2001). However, the threshold of H_2_O_2_ toxicity to rainbow trout (*Oncorhynchus mykiss*) fry and fingerlings is on the order of 10 mM (10^−2^ M) or 1 mM (10^−3^ M) H_2_O_2_, for 30 or 60 min of exposure, respectively (Arndt and Wagner 1997). Thus we conclude that the residual H_2_O_2_ in the post-reaction solution should not produce toxic effects when released into the environment if the initial phenol levels were high enough to consume the bulk of the H_2_O_2_ in the reaction. However, the residual H_2_O_2_ may rise to toxic levels for reactions with low initial phenol concentrations. In that case, the H_2_O_2_ concentration in the reaction mixture should be adjusted to assure its efficient scavenging during the incubation.

### Effect of the Weight of the Potato Pulp Inoculum and Hydrogen Peroxide Concentration on Phenol Removal

**Fig. 4:**
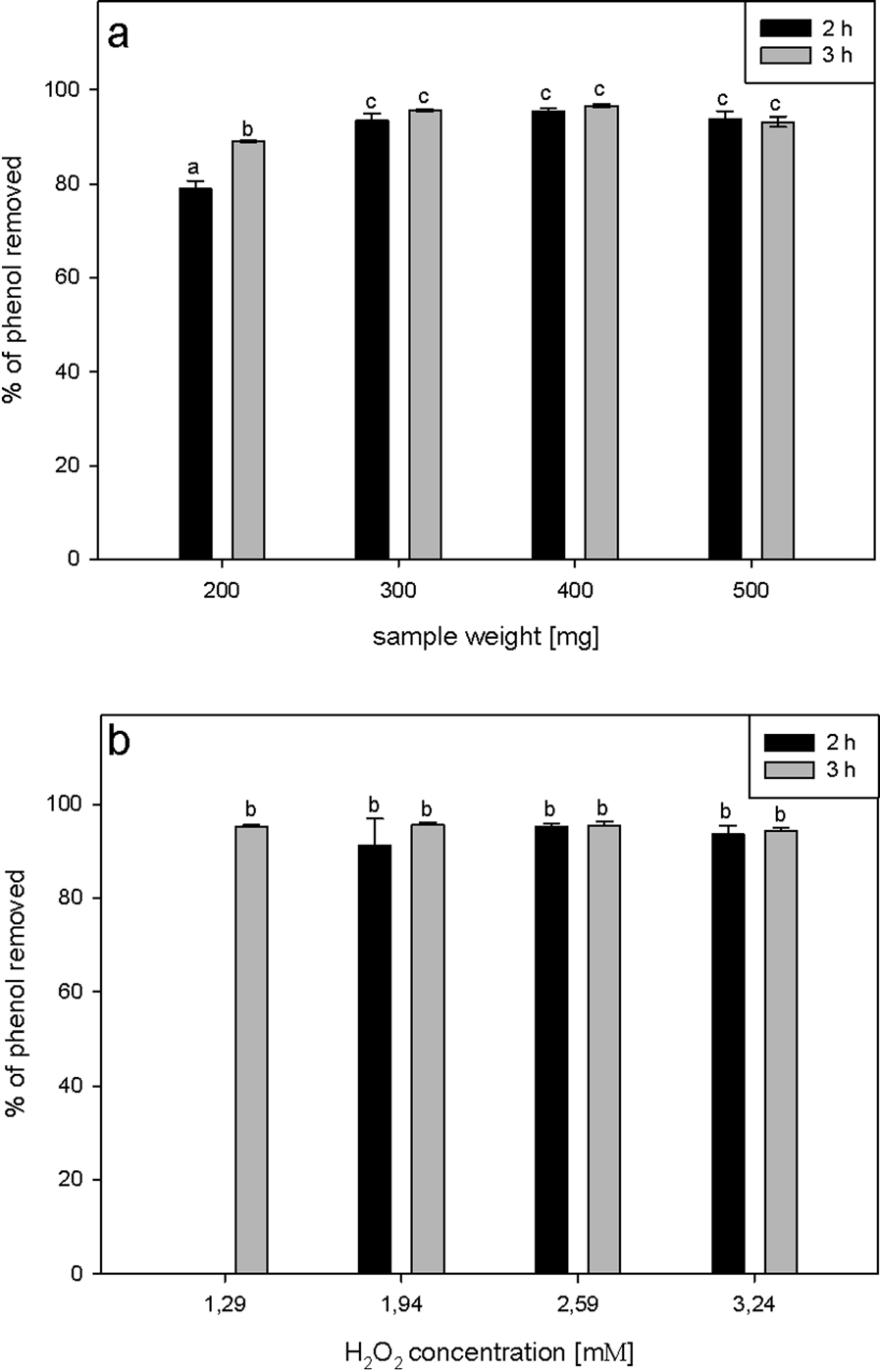
The effect of sample weight (**a**) and H_2_O_2_ concentration on phenol (phe) removal. The reactions were performed for 2 (*black bars*) or 3 (*gray bars*) hours with 1 mM phenol. Different *letters* denote significant differences at *p* ≤ 0.05

To optimize the mass of the potato pulp inoculum, we tested weights from 200 to 500 mg per 2.5 ml of reaction mixture. We observed the highest efficiency of phenol removal (from 93.2 ± 1.1 to 96.3 ± 0.2 %), for both 2 and 3 h of reaction time, when we used 300 to 500 mg of potato pulp per reaction. There were no statistically significant differences between samples from 300 to 500 mg. Supplementing the reaction mixture with more than 500 mg of pulp hampered manipulating the samples because the pulp swelled in the solution. Decreasing the weight to 200 mg still resulted in high (89 ± 0.2 % after 3 h of incubation), but statistically lower phenol removal efficiency compared to the other sample weights (Fig. 4a). The potato pulp tested displayed a higher volume of enzyme source per volume of reaction medium than was described for other systems. Naghibi et al. (2003) tested 0.6 g of *Raphanus sativus* root per 20 ml of reaction medium, but obtained only 58 % phenol removal efficiency. However, González et al. (2006, 2012) and Sosa Alderete et al. (2012) demonstrated efficiencies ranging up to 100 % phenol removal, using 0.4 g of hairy roots per 10 ml of reaction medium. We also tested a series of H_2_O_2_ concentrations to assess the effect of the oxidant concentration on phenol removal efficiency. We found over 90 % phenol removal in the solutions supplemented with all the H_2_O_2_ concentrations tested. However, with 1.29 mM H_2_O_2_, there was no phenol removal after 2 h of incubation (Fig. 4b). Removal efficiencies with 1.94, 2.59, or 3.24 mM H_2_O_2_ were comparable when measured after 2 and 3 h of incubation. However, the smallest variation in the removal efficiency, as inferred from the standard deviation values, was observed for 2.59 mM H_2_O_2_; hence, this H_2_O_2_ concentration was chosen for further experiments (Fig. 4b).

### Effect of the Reaction Mixture pH on Phenol Removal

**Fig. 5:**
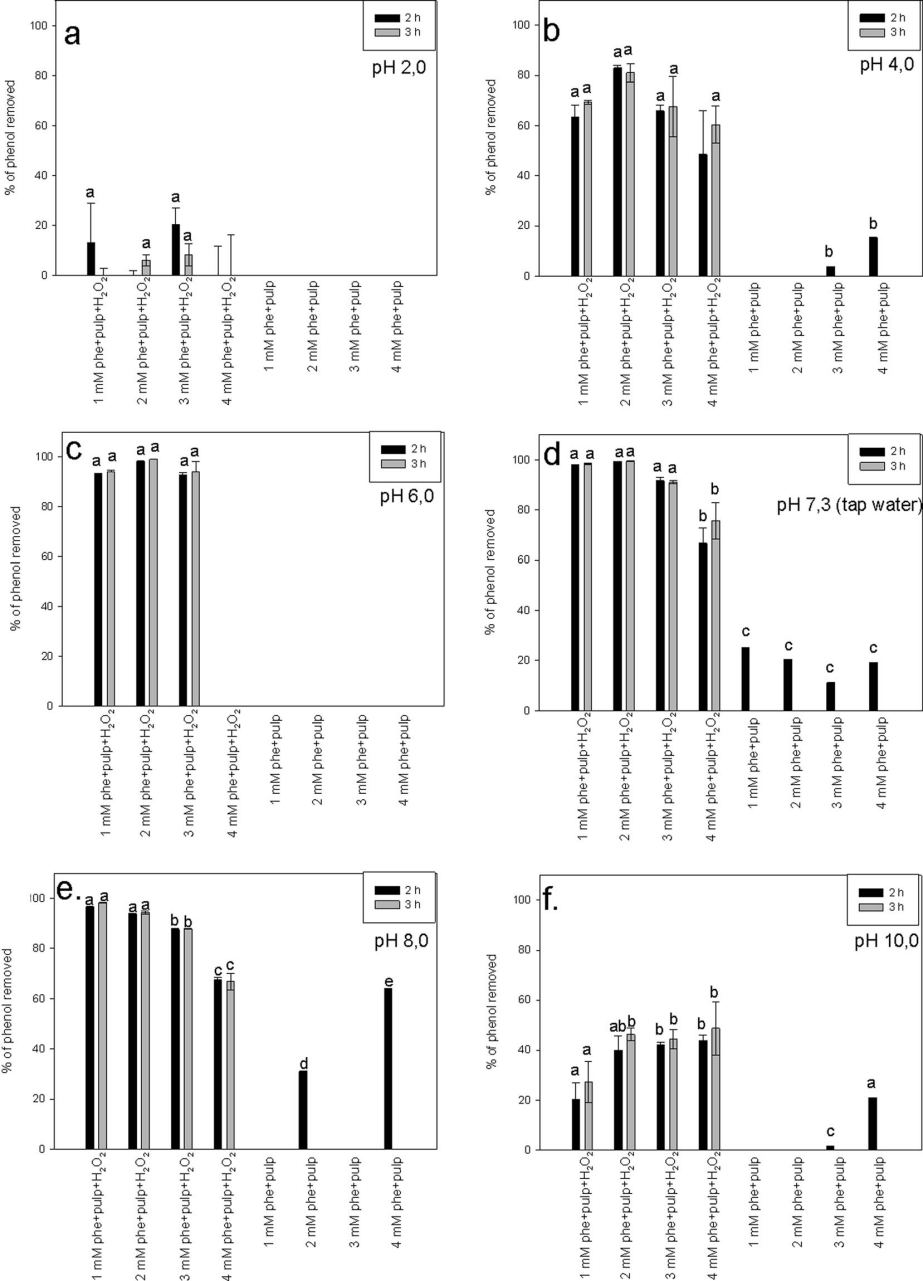
The effect of pH on the efficiency of phenol (phe) removal. Efficiency of phenol removal was assessed in assay mixtures composed of phenol solution, potato pulp inoculum, and H_2_O_2_ and in corresponding control mixtures containing phenol and potato pulp but not H_2_O_2_. The assays were carried on at pH 2.0 (**a**), pH 4.0 (**b**), pH 6.0 (**c**), pH 7.3 (tap water) (**d**), pH 8.0 (**e**), or pH 10.0 (**f**). The reactions were incubated for 2 (*black bars*) or 3 (*gray bars*) hours. Different *letters* denote significant differences at *p* ≤ 0.05

The pH range tested was from pH 2.0 to 10.0 for initial phenol concentrations from 1 to 4 mM (Fig. 5). The efficiency of phenol removal was measured after 2 or 3 h of incubation. Each time, H_2_O_2_-free controls were run along with the reactions to monitor peroxidase-independent changes in phenol concentration. At pH 2.0, the phenol was either not removed from the solution or the removal efficiency was low (Fig. 5a).

At pH 4.0, we observed better phenol removal efficiencies, with the highest reaching over 80 % for the 2 mM initial phenol concentration, whereas at the same pH, González et al. (2006) obtained phenol removal efficiency of about 45 %. Our results are similar to those of Caza et al. (1999), who found that at this pH, about 70 % of the phenol was removed from the reaction medium by soybean peroxidase. For all the phenol concentrations tested, the removal efficiencies at higher pH were higher than at pH 2.0. However, we did not observe statistically significant differences among the efficiencies of phenol removal reactions differing in their initial phenol concentrations (Fig. 5b).

At pH 6.0, a typical optimum pH for peroxidase activity, reactions resulted in very high phenol removal efficiencies, up to almost 99 % for 1, 2, or 3 mM phenol, but at a phenol concentration of 4 mM, we did not observe any phenol removal. There were no statistically significant differences in removal efficiencies between the concentration variants where phenol removal occurred (Fig. 5c). For the same pH, 1 mM phenol was removed with an efficiency of about 70 and 90 % by tomato hairy roots (González et al. 2006) and soybean peroxidase (Caza et al. 1999), respectively.

The phenol removal efficiency in the tap water (pH 7.3) as a reaction medium was 99 % for 1, 2, or 3 mM initial phenol concentrations, whereas the removal efficiency at 4 mM initial phenol concentration was 75 % after 3-h reaction (Fig. 5d). Those findings are in agreement with results obtained by Naghibi et al. (2003), who achieved over 90 % phenol removal efficiency in unbuffered tap water.

At pH 8.0, we also observed the removal of over 90 % of phenol from the treated solution (Fig. 5e). However, at higher phenol concentrations, the removal efficiencies were lower than in tap water. The highest phenol removal was observed at the 1 mM phenol concentration and reached over 96 %, whereas at this pH, hairy roots and soybean peroxidase removed about 70 and 85 % of the phenol, respectively (González et al. 2006; Caza et al. 1999). The removal efficiency gradually decreased with increasing phenol concentration to 66 % at 4 mM phenol (Fig. 5e). At pH 10, which was the highest pH tested, we observed a significant decrease in phenol removal, irrespective of the initial phenol concentration in the reaction mixture (Fig. 5f).

The results of the assays on the effect of pH show that phenol can be efficiently removed from the medium at pH 6 to 8, and the buffer may be replaced with tap water without compromising the removal efficiency. Irrespective of the pH, the efficiency of phenol removal was either significantly reduced, or did not occur, unless H_2_O_2_ was added to the reaction mixture (Fig. 5a–f). Our results are consistent with those of González et al. (2006) who reported that, using tomato hairy roots, high phenol removal efficiencies were observed in the pH range from 4 to 9, with an optimal pH of 7.5. Similarly, Caza et al. (1999) and Wu et al. (1997) observed peroxidase activity and resulting phenol removal at a wide range of pH values, from 4 to 9, and from 4 to 10, respectively, with the neutral pH as optimal. By contrast, Kalaiarasan and Palvannan (2014) observed that stabilized horseradish peroxidase displayed the highest phenol removal efficiency at pH 4.2.

### Effect of Temperature on Phenol Removal

**Fig. 6:**
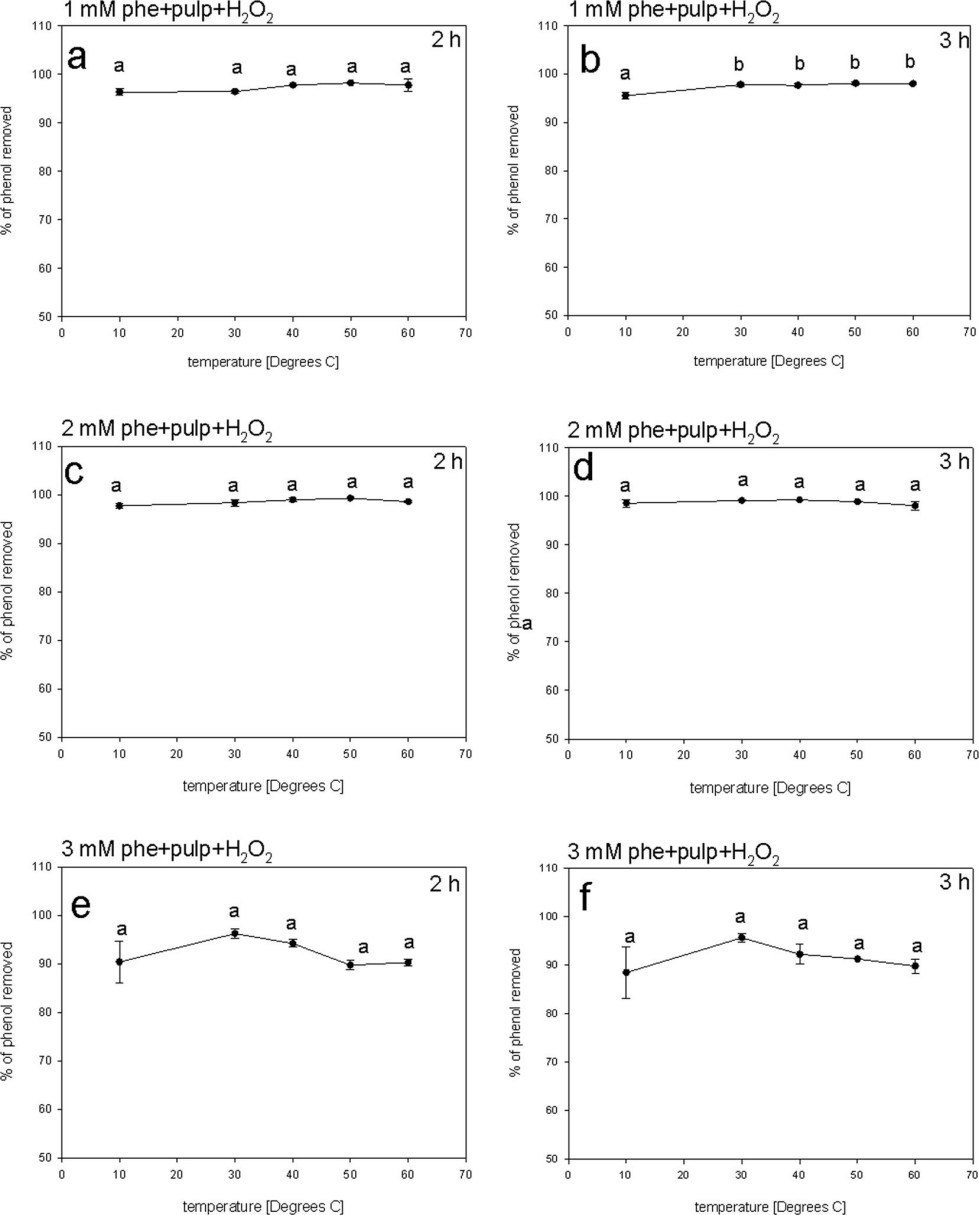
The effect of temperature on the efficiency of phenol (phe) removal. Efficiency of phenol removal was assessed in assay mixtures composed of phenol solution, potato pulp inoculum, and H_2_O_2_. The assays were incubated at different temperatures. The percentage of phenol removal was determined after 2 (**a**, **c**, **e**) or 3 (**b**, **d**, **f**) hours of incubation. The assay solutions were initially supplemented with 1 (**a**, **b**), 2 (**c**, **d**), or 3 (**e**, **f**) mM phenol. Different *letters* denote significant differences at *p* ≤ 0.05

Peroxidases usually have a wide temperature range within which their activity is maintained (González et al. 2006). Here, we studied the efficiency of phenol removal at temperatures from 10 to 60 °C. We observed that phenol removal displayed high efficiency within the tested temperature range. In the presence of 1 mM phenol, the removal efficiency did not drop below 95 %, even at the highest temperature tested (Fig. 6a, b). Similarly, the removal efficiency for 2 mM phenol was 97 % at 10 °C after 2 h of incubation and did not significantly change over the entire temperature range (Fig. 6c, d). The most distinct differences in phenol removal efficiencies at different temperatures were observed if the reaction was carried out in the presence of 3 mM phenol. In that case, at both 10 and 60 °C, we observed a slight inhibitory effect on the reaction; however, the removal efficiencies were still over 88 % and we detected no significant differences within the tested temperature range (Fig. 5e, f). For comparison, Kalaiarasan and Palvannan (2014) reported that native HRP lost its activity at 40 °C. However, the enzyme’s capacity for phenol removal was maintained at higher temperatures (65 °C) if dextran and sodium alginate were added to the reaction mixture. By contrast, González et al. (2006) observed the highest phenol removal efficiency at 40 and 60 °C, using tomato hairy roots.

### Effect of Shaking Rate on Phenol Removal

**Fig. 7:**
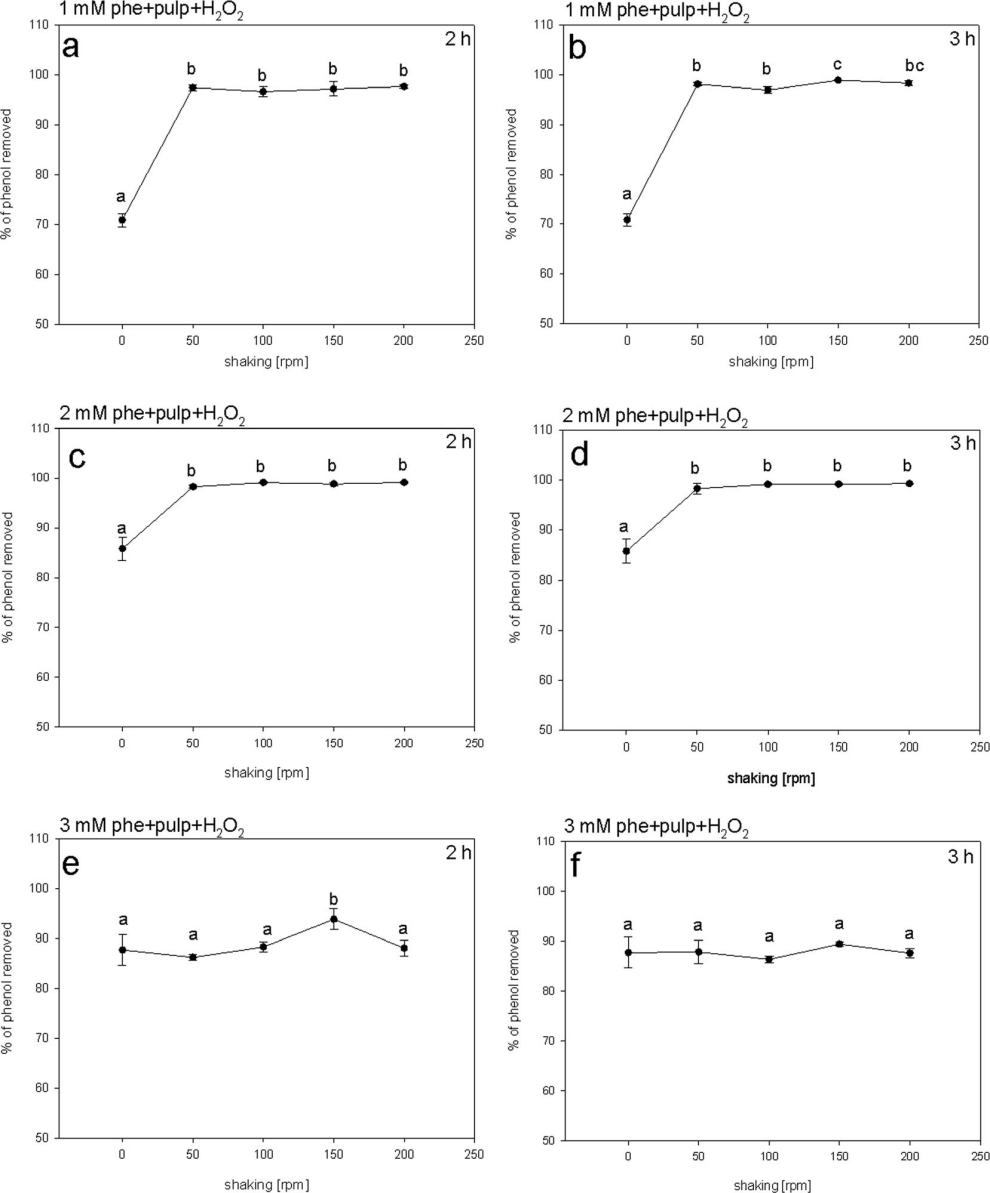
The effect of shaking rate on the efficiency of phenol (phe) removal. Efficiency of phenol removal was assessed in assay mixtures composed of phenol solution, potato pulp inoculum, and H_2_O_2_. The assays were conducted at different shaking rates. The percentage of phenol removed was determined after 2 (**a**, **c**, **e**) or 3 (**b**, **d**, **f**) hours of incubation. The assay solutions were initially supplemented with 1 (**a**, **b**), 2 (**c**, **d**), or 3 (**e**, **f**) mM phenol. Different *letters* denote significant differences at *p* ≤ 0.05

Most studies testing the ability of peroxidases to detoxify phenol solutions used shaking rates from 50 to 200 rpm to achieve optimal results (González et al. 2008, 2013; Paisio et al. 2010; Gholizadeh et al. 2013; Asadgol et al. 2014; Kalaiarasan and Palvannan 2014; Malani et al. 2013; Abdallah 2013; Angelini et al. 2014). However, to the best of our knowledge, assessing the effect of various shaking rates on the efficiency of phenol removal by peroxidase has not yet been tested as a distinct experiment. To assess the effect of shaking rate on phenol removal by potato pulp, the reaction mixtures subjected to shaking rates from 50 to 200 rpm were compared to the control, which was mixed after the reaction mixture was composed, and then left unshaken. The differences in the removal efficiency between the shaken samples compared to the control were the most obvious for the 1 mM phenol solutions. The variants that were not shaken displayed phenol removal efficiencies of 70 %, whereas shaking the samples enabled almost 100 % phenol removal within the entire range of shaking intensities (Fig. 7a, b). In the case of 2 mM phenol, the unshaken controls displayed removal efficiencies of 85 %, significantly lower than in the agitated variants. Agitating samples at 50 rpm increased phenol removal up to 98 %. The other variants also displayed removal efficiencies close to 100 % (Fig. 7c, d). After 2 and 3 h of reaction, there were no statistically significant differences in phenol removal efficiencies at shaking rates from 50 to 200 rpm. Slightly lower removal efficiencies were detected for reaction mixtures with 3 mM phenol. The unshaken controls displayed removal efficiencies of 87 %, and the efficiencies did not significantly change over the agitation rates applied, except the samples subjected to 150 rpm, after 2-h incubation, rising to over 90 % phenol removal (Fig. 7e, f).

We show that the efficiency of the removal reaction is dependent on agitating the samples, for reaction mixtures with 1 or 2 mM phenol, whereas shaking does not affect phenol removal at higher concentrations of the pollutant. Moreover, the reaction’s efficiency remained unchanged throughout the range of shaking intensities tested (Fig. 7). These finding may provide clues for the optimization of energy usage by future technology employing potato pulp for phenol removal from wastewater.

### *Lepidium sativum* Toxicity Test

**Fig. 8:**
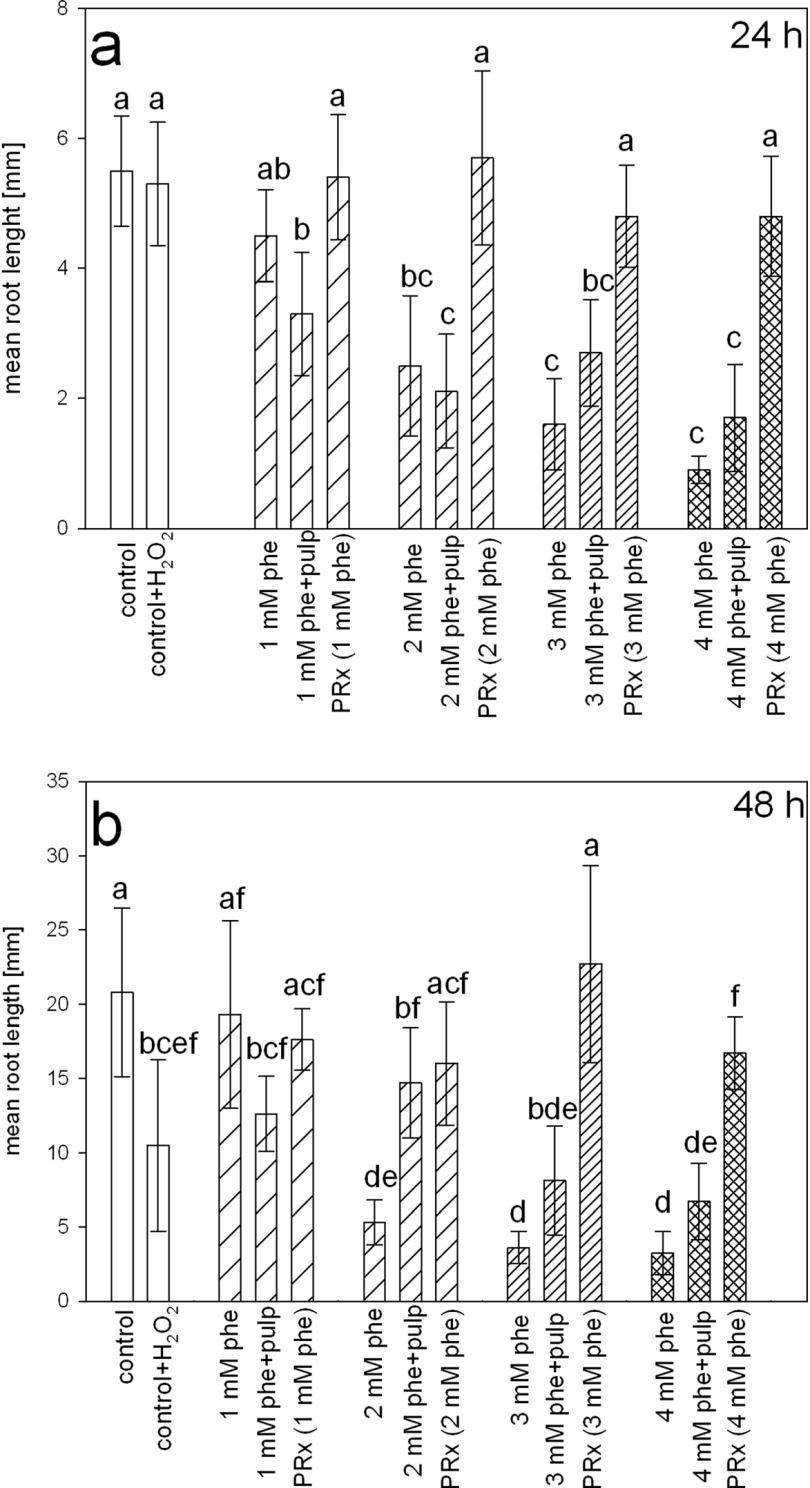
*Lepidium sativum* toxicity test. The length of the seedling root is shown. *L. sativum* seeds were treated for 24 (**a**) or 48 (**b**) hours with distilled water (control), 0.55 mM H_2_O_2_ (control + H_2_O_2_), 1–4 mM phenol (phe) solutions, phenol solution incubated with potato pulp but not provided with H_2_O_2_ (phe + pulp) or the corresponding post-reaction solution (PRx). Different *letters* denote significant differences at *p* ≤ 0.05

To find out whether the removal of phenol catalyzed by the potato pulp peroxidase reduced the toxicity of the phenol solution, we compared the root growth response of *Lepidium sativum* seedlings treated with the post-reaction mixtures to seedlings treated with unreacted phenol solution, for initial phenol concentrations of 1–4 mM. Concurrently, separate sets of seedlings were treated with H_2_O_2_-unsupplemented mixtures of phenol and potato pulp, to discriminate between the effects of peroxidase-dependent and peroxidase-independent detoxification. Additionally, to assess the toxicity of the residual H_2_O_2_, a set of seedlings was incubated with H_2_O_2_ solution at a concentration equivalent to that of the post-reaction mixture. The length of seedling roots was determined after 24 or 48 h of incubation (Fig. 8).

When the *Lepidium sativum* seedlings were incubated for 24 h in the presence of phenol, we observed progressive inhibition of seedling root growth. Treatment with 1 mM phenol did not significantly reduce seedling root length, whereas, higher phenol concentrations displayed a gradually increasing inhibitory effect on root growth. Roots of seedlings treated with H_2_O_2_-free, phenol-pulp mixture remained approximately at the same length as those incubated in the presence of unreacted phenol. By contrast, roots of seedlings treated with post-reaction solution were significantly longer than roots of seedlings treated with unreacted phenol or phenol incubated with potato pulp, being comparable to the roots of control plants, irrespective of the initial phenol concentration (Fig. 8).

Similar to the seedlings subjected to the 24-h incubation, those treated for 48 h with the phenol solutions displayed strong reductions in the root length if 2, 3, or 4 mM phenol was used. Seedlings treated with 1 mM phenol did not display statistically significant differences in root growth rate, compared to those subjected to treatment with the relevant post-reaction solution. However, there were significant reductions in root length between seedlings incubated with 1 mM phenol or the post-reaction solution, when compared to those treated with 1 mM phenol mixed with potato pulp. The reduction in the root growth, due to presence of 2–4 mM phenol, was fully abolished if phenol solution was replaced by the relevant post-reaction mixture (Fig. 8).

Seedlings grown for 48 h in the presence of a mixture of pulp and 3 or 4 mM phenol (but devoid of H_2_O_2_) produced slightly longer roots than those treated with unreacted phenol. However, the differences did not pass the significance test. In the case of seedlings incubated with 2 mM phenol-potato pulp mixture, root lengths were similar to those incubated with 2 mM post-reaction solution. Having analyzed the results of the 24 h incubation, we observed that seedling growth was not arrested due to the presence of residual H_2_O_2_. Based on that observation, we concluded that the concentration of hydrogen peroxide used had no toxic effect on *Lepidium sativum* seedlings at the initial phase of root growth (Fig. 8a). However, the 48-h exposure to this reagent resulted in a significant decrease in root length. This suggests that prolonged presence of oxidant concentrations similar to those remaining in the post-reaction mixtures may have a toxic effect. However, any growth inhibition was not observed if the post-reaction mixture was used instead of the H_2_O_2_ solution (Fig. 8). This finding could be explained by the scavenging of the residual H_2_O_2_ at the expense of the residual phenol by secretory peroxidase of *Lepidium sativum* during seedling incubation.

Our results are consistent with those obtained by Angelini et al. (2014), who demonstrated, using *Lactuca sativa* seedlings, that the toxicity caused by 2,4-dichlorophenol was completely abolished after removal of the pollutant with *Brassica napus* hairy roots. Paisio et al. (2010) reported that *Rhinella arenarum* embryos treated with post-reaction solution from rapeseed hairy roots displayed significantly reduced mortality for initial phenol concentrations from 1 to 2.65 mM, compared to the corresponding concentrations of unreacted phenol. PEG 3350 in the assay medium significantly enhanced the reduction of phenol toxicity during the phenol removal reaction (Paisio et al. 2010). The results of toxicity tests with onion (*Allium cepa*) bulbs performed by González et al. (2012) show that phenol removal by tomato hairy root peroxidase significantly reduced the toxicity of the solution originally supplemented with 2.65 mM phenol. Onion bulbs treated with phenol post-reaction solutions displayed an increase of 34 % in root length, compared with the phenol solution of the same concentration (González et al. 2012).

### Phenol Removal from Industrial Effluent

**Fig. 9:**
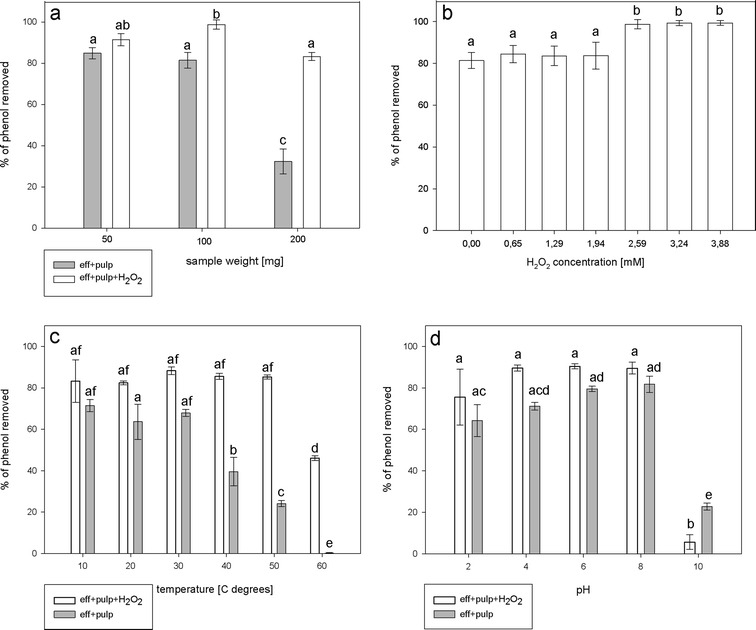
Phenol removal from industrial effluent. Different conditions for phenol removal were tested. The reactions were performed for 2 h with 50–200 mg of potato pulp and supplemented with 2.59 mM H_2_O_2_ (**a**). The effect of H_2_O_2_ concentration on phenol removal (**b**). The effect of temperature on phenol removal (**c**). The effect of pH on phenol removal (**d**). eff + pulp + H_2_O_2_ = reaction mixture composed of industrial effluent, potato pulp and supplemented with H_2_O_2_, eff + pulp = industrial effluent incubated with potato pulp, but devoid of H_2_O_2_. Different *letters* denote significant differences at *p* ≤ 0.05

The phenol concentration in the samples of the industrial effluent used in this study varied between 0.02 and 0.1 mM. Since the phenol concentrations in the industrial effluent were much lower compared to the synthetic wastewater prepared in the laboratory, we anticipated that smaller inocula of potato pulp will be required for phenol removal. Therefore, 50, 100, or 200 mg of potato pulp samples were assessed for their ability to decrease phenol content in the industrial wastewater. It was determined that using 100 mg of potato pulp resulted in the highest phenol removal from the industrial effluent. In contrast to the synthetic wastewater, up to 80 % of phenol was removed by potato pulp from industrial wastewater in the absence of H_2_O_2_ (Fig. 9a). This finding may suggest effective phenol sorption to pulp solids. Increasing H_2_O_2_ concentration until 1.94 mM did not affect removal efficiency. However, 2.59 mM H_2_O_2_ or higher concentrations enhanced the removal efficiency (Fig. 9b).

The efficiency of phenol removal in H_2_O_2_-unsupplemented medium progressively decreased under incubation temperature of 40 °C or higher. However, the removal process was still effective in H_2_O_2_-supplemented samples (Fig. 9c) suggesting that peroxidase-based phenol removal was less susceptible to high temperature than in H_2_O_2_-free controls. We also observed, that the reaction could be conducted over a wide range of the pH range. The significant decrease in phenol removal efficiency was observed only when the reaction was conducted at pH 10 (Fig. 9d).

## Conclusion

The bottleneck for systems using peroxidases for the decontamination of phenol-polluted wastewater is the inaccessibility of a low-cost enzyme source, which would satisfy the demands of large-scale remediation. The use of potato pulp for remediation may help to overcome this difficulty, since potato starch factories produce hundreds thousands of tons of this material every year as an industrial waste product. Potato pulp provides a good alternative to purified peroxidases, such as HRP, which are expensive and prone to inactivation, and to hairy root cultures, which—while ensuring good removal efficiencies—are also costly, due to the high cost of culture media. The present work opens the possibility of further research aimed at scaling-up the process and construction of bioreactors adapted to large-scale decontamination of phenol-polluted wastewater with potato pulp.
